# Automated redaction of names in adverse event reports using transformer-based neural networks

**DOI:** 10.1186/s12911-024-02785-9

**Published:** 2024-12-23

**Authors:** Eva-Lisa Meldau, Shachi Bista, Carlos Melgarejo-González, G. Niklas Norén

**Affiliations:** Uppsala Monitoring Centre, Uppsala, Sweden

**Keywords:** De-identification, Data anonymization, Pharmacovigilance, Domain adaptation, Adverse drug reaction reporting systems, Medical language processing

## Abstract

**Background:**

Automated recognition and redaction of personal identifiers in free text can enable organisations to share data while protecting privacy. This is important in the context of pharmacovigilance since relevant detailed information on the clinical course of events, differential diagnosis, and patient-reported reflections may often only be conveyed in narrative form. The aim of this study is to develop and evaluate a method for automated redaction of person names in English narrative text on adverse event reports. The target domain for this study was case narratives from the United Kingdom’s Yellow Card scheme, which collects and monitors information on suspected side effects to medicines and vaccines.

**Methods:**

We finetuned BERT – a transformer-based neural network – for recognising names in case narratives. Training data consisted of newly annotated records from the Yellow Card data and of the i2b2 2014 deidentification challenge. Because the Yellow Card data contained few names, we used predictive models to select narratives for training. Performance was evaluated on a separate set of annotated narratives from the Yellow Card scheme. In-depth review determined whether (parts of) person names missed by the de-identification method could enable re-identification of the individual, and whether de-identification reduced the clinical utility of narratives by collaterally masking relevant information.

**Results:**

Recall on held-out Yellow Card data was 87% (155/179) at a precision of 55% (155/282) and a false-positive rate of 0.05% (127/ 263,451). Considering tokens longer than three characters separately, recall was 94% (102/108) and precision 58% (102/175). For 13 of the 5,042 narratives in Yellow Card test data (71 with person names), the method failed to flag at least one name token. According to in-depth review, the leaked information could enable direct identification for one narrative and indirect identification for two narratives. Clinically relevant information was removed in less than 1% of the 5,042 processed narratives; 97% of the narratives were completely untouched.

**Conclusions:**

Automated redaction of names in free-text narratives of adverse event reports can achieve sufficient recall including shorter tokens like patient initials. In-depth review shows that the rare leaks that occur tend not to compromise patient confidentiality. Precision and false positive rates are acceptable with almost all clinically relevant information retained.

**Supplementary Information:**

The online version contains supplementary material available at 10.1186/s12911-024-02785-9.

## Background

Understanding a patient’s experience from a written record requires detailed information on the clinical course of events, any differential diagnosis, along with patient-reported reflections and observations. While some of this can be captured in structured data fields, nuances and details can sometimes only be conveyed in narrative form via free-text fields. Pharmacovigilance is the science and activities relating to the detection, assessment, understanding and prevention of adverse effects or any other medicine/vaccine related problem [1, 2]. Its cornerstone is adverse event reports communicating suspected harm from medicines in individual patients [3, 4]. Their unique value is the information captured specifically to support causality assessment, much of which is communicated in narrative form [5, 6]. A major limitation today is the difficulty for different organisations to share complete reports without risking compromising patient confidentiality. The degree to which different pieces of information may allow patients to be identified varies [7]. Some, like names, ID numbers and addresses may be direct identifiers of individuals. Others like ages, dates and initials may, together with other information, enable re-identification indirectly.

Automated recognition and redaction of personal identifiers in free text can enable organisations to share data while preserving privacy. In this study, we limit the scope and focus on detecting person names in narratives written in English. Names are more difficult for automated systems to recognise than other direct identifiers because context needs to be considered. In the two statements: “The patient was diagnosed with Stevens-*Johnson* syndrome.” And “Mr *Johnson* a 60-year-old patient with Type II diabetes.” *Johnson* relates to a medical condition in the first, while to a patient’s last name in the second. This occurs when medical terms are named after people, so called medical eponyms, or when common English words used in medicine appear as first or last names of people. Their disambiguation is challenging [8] and requires advanced language models which can generalise to new context better than rules. Automated de-identification has evolved from rule-based methods [9], through hybrids including e.g. Conditional Random Fields [10], to deep neural networks including recurrent neural networks [11] and transformer models [12, 13]. Neural networks and specifically transformer models like BERT (Bidirectional Encoder Representations from Transformers) have shown great value in Natural Language Processing (NLP) advancing the state-of-the-art [14–17].

Neural networks like BERT are pre-trained on large text corpora and then fine-tuned to the target domain [18, 19], for example using publicly available, annotated data sets [20]. For de-identification, the 2014 i2b2/UTHealth Corpus dataset provides electronic discharge summaries and correspondences between medical professionals, with annotated personal identifiers [7]. However, adverse event reports are different in nature, and their structure, content, and language may vary depending on whether a patient or health professional wrote the report. The prevalence of personal identifiers may also vary, depending on the reporter’s awareness of data protection considerations and legislation. Thus, the development of a de-identification method of adverse event reports requires domain-specific data and evaluation framework. Clinical utility such as readability and preservation of clinal context is also an important consideration in evaluation of de-identification methods. To address this, some studies have evaluated machine learning models trained on redacted vs. original data [21, 22], while others have manually evaluated impact on readability [23].

The aim of this study was to develop and evaluate a method for automated redaction of names in case narratives on adverse event reports in English using a BERT model fine-tuned for the task. Specifically, we sought to assess what recall of names can be achieved and how much clinically relevant information may be collaterally masked. The study used data from the United Kingdom’s Yellow Card scheme [24] and, to our knowledge, is the first evaluation of a de-identification method in adverse event reports. A poster summarising an early version of this study was presented at the 2022 annual meeting of the International Society of Pharmacovigilance [25].

## Methods

**Fig. 1:**
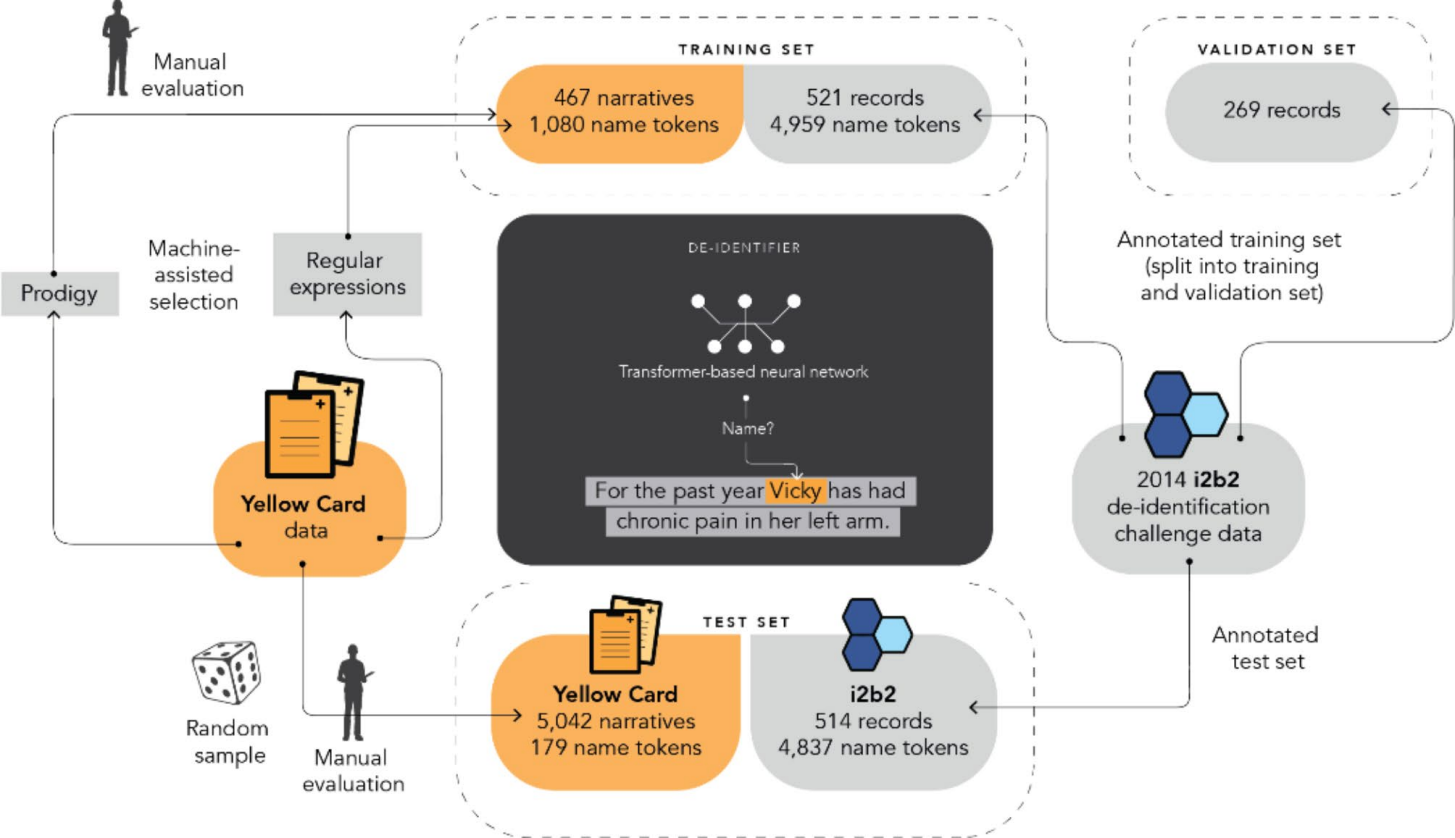
Overview of the de-identification method and dataset preparation

Our de-identification method was developed and evaluated using data from the i2b2 2014 dataset and adverse event reports from the UK Yellow Card scheme. An overview of the method and the dataset preparation is depicted in Fig. 1 and described in this section.

### Data

#### The 2014 i2b2/UTHealth corpus

One of our two sources of data for development of the proposed method was the i2b2 2014 de-identification challenge dataset. It consists of 1,304 annotated and anonymised discharge summaries from a collection of electronic medical records of 296 patients. Three subcategories in this dataset represent person names: doctor, patient, and username. This dataset is a well-studied benchmark for the de-identification of medical text. Consequently, there is significant literature and methods evaluated on this dataset [6, 11, 12, 26]. The corpus is distributed as a training set of 790 records, and a test set of 514 records [7].

### Yellow card data

As a second source of data for development, as well as for evaluation, we used reports from the UK Yellow Card scheme received via the Yellow Card website^1^, Coronavirus Yellow Card reporting site^2^ and Yellow Card mobile reporting apps^3^^,^^4^. The data consisted of all 124,420 narratives received through these channels before 25th of November 2020. The narratives were extracted before any manual data processing steps, in the same version as sent to the MHRA’s case processing system.

### Training and validation sets

Our training set for fine-tuning BERT combined data from the i2b2 and Yellow Card data sets.

From the annotated i2b2-provided training set, we incorporated 521 records into our training set, and used the remaining 269 records as our validation set. Our training set included 3,030 instances of names across the three sub-categories: doctor (1,932), patient (879), and username (219). These instances consisted of 4,959 NAME tokens after tokenisation, of which 544 were three or less characters long. In comparison, there were 359,214 NON-NAME tokens.

From the Yellow Card data set, we set aside 74,731 narratives (60%) for possible inclusion in our training set. Due to a low prevalence of names in the Yellow Card data, we deployed machine-assisted annotation where two independent methods were used to identify narratives more likely to contain person names. The first used the Prodigy software^5^ (version 1.10), which, based on a set of common first and last names, identified semantically similar words. It then iteratively applied and refined a predictive model selecting narratives for subsequent manual annotation, using active learning. Initials proved harder to capture using this strategy. Thus, we identified additional narratives for manual annotation using a set of regular expressions based on the context seen around names identified with Prodigy. For a more detailed description of the machine-assisted annotation process see S1 in the supplementary materials.

In total, 467 narratives from the Yellow Card data were annotated for our training set. 416 of these were identified by Prodigy and 56 by regular expressions, including an overlap of 5 narratives identified by both approaches. This included 299 narratives with at least one name instance and a total of 1,080 NAME tokens (267 of which consisted of three or less characters) and 79,068 NON-NAME tokens (33,165 of which consisted of three or less characters).

### Test sets

Our primary test set was based on Yellow Card data. 5,042 narratives were randomly selected from nearly 50,000 narratives held out from the development of our training set. 71 of these narratives included a name with a total of 179 NAME tokens. For the test set four annotators were provided with subsets of the narratives and annotated all names according to our annotation guideline (see S2 in supplementary materials).

The i2b2 test set of 514 records was held out and used for secondary performance evaluation. It included 2,883 instances of names across the three sub-categories: doctor (1,912), patient (879), and username (92). It comprised in total 4,837 NAME tokens and 263,272 NON-NAME tokens.

### De-identification method

The core component of our de-identification method was a fine-tuned BERT binary classifier. We also considered an ensemble method incorporating a rule-based classifier. The ensemble consumed binary labels from the rule-based and BERT classifiers and performed an OR operation to predict the final binary label, i.e., predicting a NAME if either of the classifiers predicts this label and only otherwise NON-NAME.

The BERT model for token classification was fine-tuned on the training set and predicted a binary NAME or NON-NAME label for each token in the narratives. We used *bert-base-uncased* from *huggingface* (version 4.12.3) [27] with *Tensorflow* (version 2.7.0), which had been pretrained on unpublished books from *BookCorpus* and text from the *English Wikipedia* [16]. When predicting the NON-NAME label, the predicted score for NON-NAME had to be above a specified threshold. Using the validation data, we identified the optimal number of epochs, classification threshold, learning rate and fine-tuned the BERT model for 5 epochs using the categorical cross entropy loss function, a threshold of 0.9, and a learning rate of $\:{1e}^{-5}$ with the *Adam* optimizer. S3 in the supplementary materials contains excerpts from the code outlining how the BERT model was trained.

Since BERT can only accept 512 tokens in each sequence, we split longer narratives into shorter sequences and ran inference on them independently.

#### Complementary rule-based classifier

The rule-based classifier captured common patterns using regular expressions. These patterns were based on the i2b2 training dataset and hand engineered.

1. Between one and three words following a salutation (e.g., “dr.” or “doctor”).
2. Between one and three words following a label (e.g., “name:”, “patient:”).
3. Between one and three words before a title (e.g., “m.d.”).

The number of words captured is dependent on capitalisation. A detailed description of the rules is given in S4 in the supplementary materials.

### Evaluation

#### Overall performance evaluation

We treated de-identification as a binary token classification problem and calculated precision, recall and F1 scores for the recognition of NAME tokens. As a complement, we computed false positive rates to measure what proportion of NON-NAME tokens would unnecessarily be masked by the de-identification method, negatively impacting the utility of the redacted narratives.

**Fig. 2:**
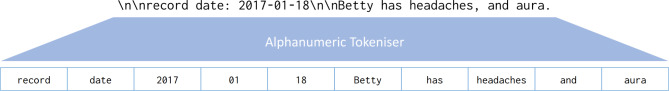
Tokens delimited by the simple alphanumeric tokeniser

Evaluation was performed at a token level using a simple alphanumeric tokeniser which defined a token as a sequence of consecutive alphanumeric characters (Fig. 2).

Metrics were calculated in what we call “covering” mode, where the NAME token flagged by the system must completely encapsulate the annotated tokens in the gold standard, and where encapsulation of NON-NAME tokens was accepted if the NAME token was also completely covered. Any partially covered NON-NAME tokens would however be counted as false positives.

To understand performance of our method on possible initials and full names, we calculated the evaluation metrics separately on tokens of length > 3, called *long tokens,* and tokens of length ≤ 3, called *short tokens*.

#### In-depth evaluation

A domain expert (co-author CM) performed an in-depth performance evaluation, following a pre-established evaluation guideline developed for this purpose (see S5 in supplementary materials). He was at that point not familiar with detailed design of the de-identification method.

All false negatives in the Yellow Card test data (annotated NAMES not flagged by the BERT de-identification method), were classified into three categories based on the nature of the names and additional information in the narrative (for example classifications, see S5 in supplementary materials):

- Directly identifiable: identification likely from leaked NAME token alone.
- Indirectly identifiable: identification likely from leaked NAME token together with additional information in the narrative.
- Non-identifiable: identification unlikely.

Narratives with false positive flags in the Yellow Card test data (tokens flagged by the de-identification method corresponding to NON-NAMES according to the annotation) were reviewed for collateral masking of clinically relevant information. First, the domain expert assessed whether clinically relevant information appeared to be missing from redacted narratives. Subsequently, the redacted text was revealed, and a similar assessment was made. Examples of clinically relevant information included details on adverse events, diagnoses, treatments, or any other information that shed light on the clinical course of the events (for more details, see S5 in supplementary materials).

## Results

### Overall performance evaluation

**Fig. 3:**
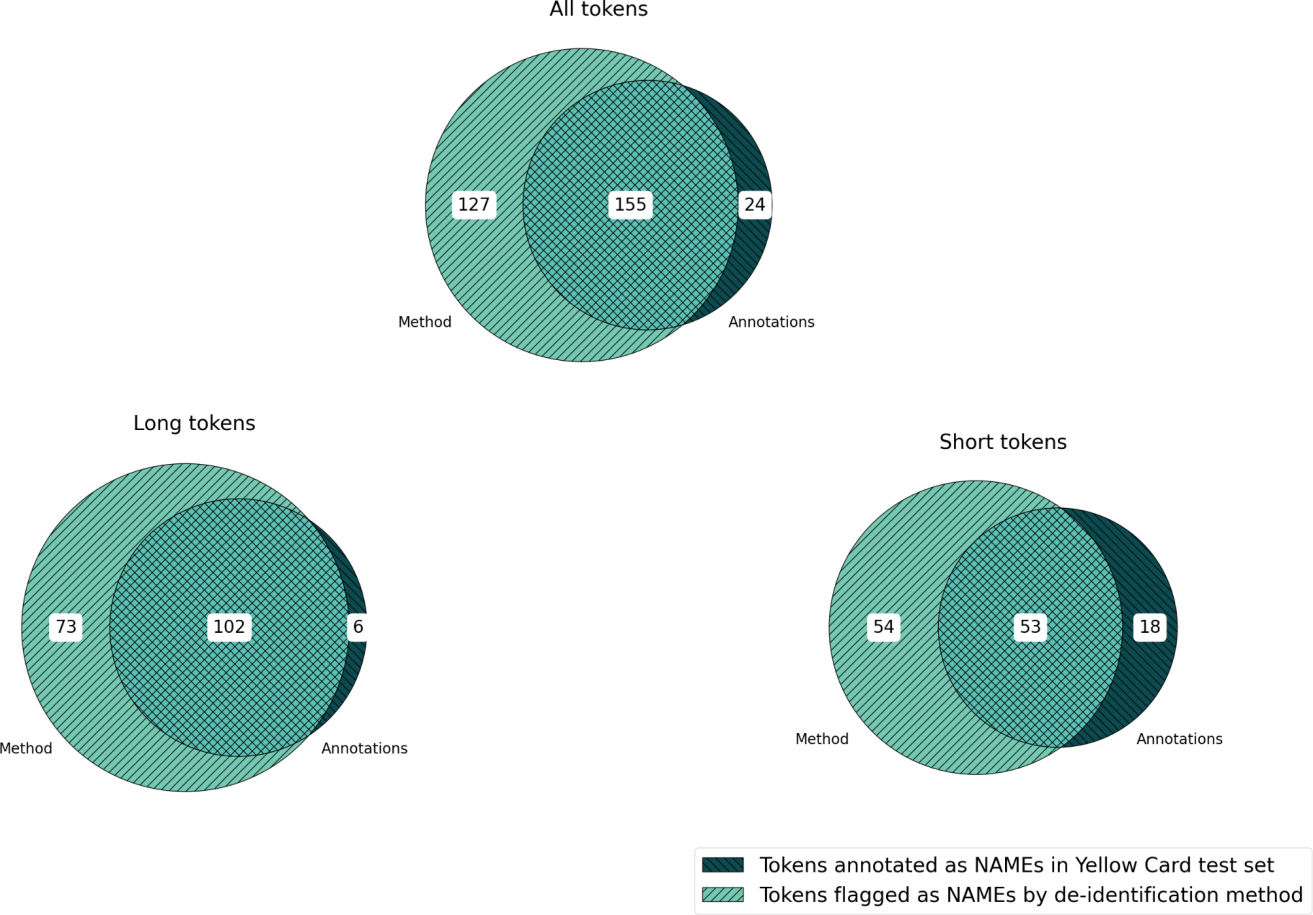
Venn diagram showing the cross-classification of tokens in the Yellow Card test data

On the 5,042 case narratives in the Yellow Card test data, our de-identification method *recalled* 87% of all annotated NAME tokens, while incorrectly flagging 0.05% NON-NAME tokens (*false positive rate*). *Precision* was 55%. The recall of longer NAME tokens was 94% and precision 58%. For shorter NAME tokens, the recall was 75% and precision 50%. For more details including F1 scores and absolute frequencies, see Table 1; Fig. 3.

**Table 1:** Performance of proposed de-identification method on Yellow Card test data

	Performance in YC test data:				Tokens in YC test data:	
Token length	Precision	Recall	F1	False positive rate	NAMES	NON-NAMES
All	55%	87%	67%	0.05%	179	263,272
Long (> 3)	**58%**	**94%**	**72%**	**0.04%**	108	162,582
Short (≤ 3)	50%	75%	60%	0.05%	71	100,690

**bold**: best score, **YC**: Yellow Card

**Fig. 4:**
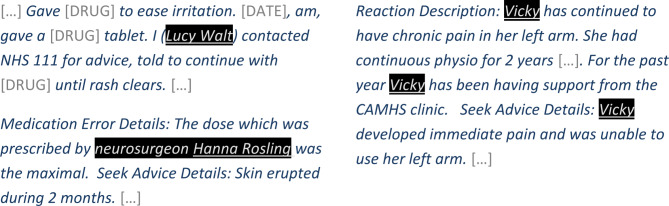
Example narratives where the NAME tokens were correctly flagged by the de-identification method (black background indicating NAME tokens flagged by the model, underline indicating NAME token annotations). N.B. All NAME tokens and personal identifiers are surrogates for similar entities in the original narratives. Drug names and dates have been replaced with placeholders

Figure 4 shows examples of NAME tokens correctly flagged by our method, in narratives from the Yellow Card test data. Please note that while the examples in this paper are real adverse event reports, all personal identifiers such as names, location, dates, and ages have been manually replaced with surrogates for inclusion in this paper.

#### Performance of an ensemble method including rules

**Fig. 5:**
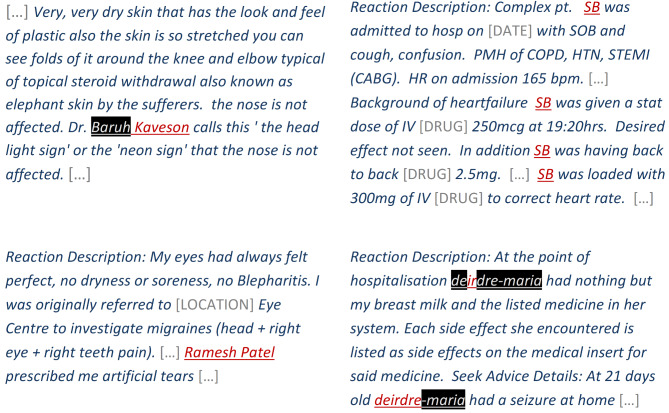
Narratives with NAME tokens not flagged by our method (black background indicating NAME tokens flagged by the model, underline indicating NAME token annotations). ‘Ramesh Patel’ was classified as a direct identifier, ‘Kaveson’ was classified as an indirect identifier, and ‘SB’ and ‘deirdre’ were classified as not enabling re-identification in the context of the narratives. N.B. All NAME tokens and personal identifiers are surrogates for similar entities in the original narratives. Drug names and medical facility names have been replaced with placeholders

Table 2 shows the performance of the ensemble combining BERT with hand-engineered rules. While precision decreases from 55 to 26% (increasing the false positive rate to 0.17%) for a modest increase in recall, the ensemble recognised one of the surnames leaked by the BERT model (see Fig. 5, top left).

**Table 2:** Performance of different components of de-identification method when applied separately on Yellow Card test data

	Performance in YC test data:					Tokens in YC test data:	
Token length	**Component**	**Precision**	**Recall**	**F1**	**False positive rate**	**NAMES**	**NON-NAMES**
All	**BERT**	**55%**	87%	**67%**	**0.05%**	179	263,272
	**BERT + rules**	26%	**88%**	40%	0.17%	179	263,272
	**Rules alone**	13%	26%	17%	0.12%	179	263,272
Long (> 3)	**BERT**	**58%**	94%	**72%**	**0.04%**	108	162,582
	**BERT + rules**	32%	**96%**	48%	0.14%	108	162,582
	**Rules alone**	20%	31%	24%	0.08%	108	162,582
Short (≤ 3)	**BERT**	**50%**	**75%**	**60%**	**0.05%**	71	100,690
	**BERT + rules**	19%	**75%**	30%	0.23%	71	100,690
	**Rules alone**	6%	17%	9%	0.17%	71	100,690

**bold**: best score, **YC**: Yellow Card

#### Runtime

Experiments were run on Intel i9-9880 H @ 2.30 GHz with 8 cores, 16 logical processors, 16GB RAM, NVIDIA Quadro T2000 GPU with 4GB dedicated and 8GB shared memory. The total runtime for all 5,042 narratives in the test set was 14 min – an average of 0.17 s per narrative.

### In-depth evaluation

There were 71 narratives with NAME annotations in the Yellow Card test data out of which 13 contained at least one leaked NAME token (recall of 82% on a *narrative-level*) after de-identification with the BERT de-identification method. Considering shorter and longer NAME tokens separately, we saw a 91% narrative-level recall for longer tokens (39 of 43 narratives) and 76% for shorter tokens (28 of 37 narratives).

According to domain expert review, false negative errors involved a *direct identifier* in one narrative (see Fig. 5 with leaked name corresponding to *Ramesh Patel*), an *indirect identifier* in two narratives (see Fig. 5 for one example with leaked name corresponding to *Kaveson*), and information that would not enable re-identification in the remaining 10 narratives (see examples in Fig. 5 with leaked initials corresponding to *SB* and leaked part of name corresponding to *deirdre*).

In three of the narratives with leaks, it is doubtful that the leaked text truly refers to person names (for example “Person A”) but we classified them as names in the gold standard to be on the safe side.

**Fig. 6:**
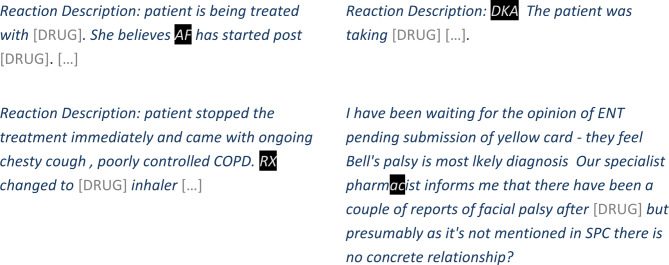
Narratives with NON-NAME tokens incorrectly flagged by the method (black background indicating NAME tokens flagged by the model, underline indicating NAME token annotations). For the two narratives to the left, the redacted text was suspected to be clinically relevant; for the two narratives on top, the redacted text was classified as clinically relevant once revealed. N.B. All NAME tokens and personal identifiers are surrogates for similar entities in the original narratives. Drug names have been replaced with a placeholder

Our de-identification method flagged one or more tokens as suspected names in 3% of the 5,042 narratives in the Yellow Card test data, leaving 97% of the narratives untouched. 63 of the touched narratives contained at least one true positive and 156 narratives contained at least one false positive. The domain expert classified 22% of the narratives with false positives as likely to be missing clinically relevant information because of the de-identification, before revealing the redacted text. After revealing the redacted text, the corresponding proportion was 20%. All combined, we would thus expect clinically relevant information to be removed in around 1 in 160 narratives. Examples of NON-NAME tokens incorrectly flagged by the method are provided in Fig. 6.

### Impact of training data on performance in yellow card test data

Table 3 shows the performance of the method on the Yellow Card test data when BERT was fine-tuned exclusively on training data from i2b2. Fine-tuning BERT on the Yellow Card training data alongside i2b2 data substantially improved recall from 66 to 87%. The increase in recall came at the cost of slightly decreased precision (from $\:59 \to 55\%$) and slightly increased false positive rate (from $\:0.03 \to 0.05\%$). The entire improvement in performance derived from better ability to flag short NAME tokens; the performance for long tokens was essentially unchanged.

**Table 3:** Impact on performance of choice of training data on performance in Yellow Card test data

		Performance in YC test data:				Tokens in YC test data:		
Token length	**Training data**	**Precision**	**Recall**	**F1**	**False positive rate**	**NAMES**	**NON-NAMES**	
All	**i2b2**	**59%**	66%	62%	**0.03%**	179	263,272	
	**i2b2 + YC**	55%	**87%**	**67%**	0.05%	179	263,272	
Long (> 3)	**i2b2**	**59%**	**95%**	**73%**	**0.04%**	108	162,582	
	**i2b2 + YC**	58%	94%	72%	**0.04%**	108	162,582	
Short (≤ 3)	**i2b2**	**63%**	21%	32%	**0.01%**	71	100,690	
	**i2b2 + YC**	50%	**75%**	**60%**	0.05%	71	100,690	

**bold**: best score, **YC**: Yellow Card

### Performance in i2b2 test data

As a benchmark, Table 4 shows the performance of the method on the i2b2 test set, when fine-tuned only on i2b2 training data compared to when fine-tuned on Yellow Card training data alongside i2b2 training data, as in our main analysis. These are the results for flagging names regardless of their i2b2 subcategory. The performance was similar, with a slight advantage for fine-tuning exclusively on the i2b2 data.

**Table 4:** Performance in i2b2 test data

	Performance in i2b2 test data:				Tokens in i2b2 test data:	
Training data	**Precision**	**Recall**	**F1**	**False positive rate**	**NAMES**	**NON-NAMES**
i2b2	**98.7%**	**97.7%**	**98.2%**	**0.018%**	4837	335,421
i2b2 + YC	**98.7%**	97.2%	97.9%	0.019%	4837	335,421

**bold**: best score

### Sensitivity analysis

The following person names were all correctly flagged by the method when inserted into the narrative displayed in Fig. 5 instead of the real name of Indian origin: all of this paper’s co-authors’ names; the following names of Indian origin ‘Ramesh Patel’, ‘Ramesh [real last name]’, and ‘[real first name] Patel’; and the common English names ‘John Smith’ and ‘Jane Smith’. The only error (other than for the real name itself) was observed for ‘John Smith’, where the method flagged ‘Smith’ but not ‘John’.

When inserting the real name of the narrative in Fig. 5 corresponding to *Ramesh Patel* into a narrative in Fig. 4 (replacing *Hanna Rosling*), it was correctly flagged by the method.

## Discussion

A fine-tuned transformer-based neural network can recognise and mask a vast majority of names and most initials while leaving most of the other information untouched. Qualitative evaluation shows that the rare leaks that occur tend not to make cases identifiable. Fine-tuning to adverse event reports was crucial in reaching good performance. The annotation guideline and qualitative evaluation framework proposed in this study can serve as a base for future performance evaluation for de-identification of medical case descriptions.

As long as the de-identified narratives are only shared with trusted parties within pharmacovigilance aiming to support safe use of medicines, we believe that this performance is already acceptable for routine use. If necessary, to further decrease the risks of leaks, the system could flag narratives for human review if they contain any suspected names, exploiting the clustered appearance of personal identifiers. This would reduce the number of narratives to be reviewed, freeing up time for other tasks. To use the method in practice, the names will also need to be removed or replaced. Covering the text with a placeholder such as “NAME” can be an option that is preferable to just removing the tokens for the purpose of readability. Alternatively, replacing with a a different, surrogate name can provide additional protection since leaked names will be hiding in plain sight [28]. However, in this setting, it would likely cause concern with the readers of redacted narratives and would be difficult to explain to all potential recipients. Studies on the impact of de-identification on downstream NLP tasks [8, 29] found no drop in performance unless for low precision de-identification, suggesting that the redaction of names will likely not prevent the development of effective models for the redacted narratives.

There were only 108 long NAME tokens in the Yellow Card test data. From a method evaluation perspective, this is a challenge and a limitation. The only full name not flagged as a suspected personal identifier was of Indian origin, but from the available data, we cannot know whether recall varies systematically with the nature of the names. Due to the complexity and opacity of BERT, we analysed this via experimentation and data manipulation. The results suggest that the observed leak was caused by a combination of the name and the context in which it appears. Limited explainability is a general concern with deep neural networks. In view of the low error rate, it may be less important to understand the inner workings of the method here, but clarity around the characteristics of the training and test data and the concrete examples of correct and incorrect classifications are necessary to assess generalisability and algorithmic fairness.

Our machine-assisted annotation strategy, using two independent methods to identify narratives more likely to contain person names, increased the rate of NAME tokens to more than 2 per narrative in the Yellow Card training set compared to 0.04 per narrative in the test set. The primary risk is that names which are more difficult to recognise would be under-represented in training data, leading to poor performance in the general case. The inclusion of i2b2 records in training may have helped mitigate this.

While for many years most state-of-the-art methods in de-identification used LSTM-CRF (Long Short-Term Memory with Conditional Random Fields) architectures [11, 30], recently, BERT has been assessed both in embedding layers and as stand-alone models [31–33] and other published methods are based on ensembles [10, 12]. State-of-the-art methods report a recall above 96% for recognising specific name categories in i2b2 test data [11, 31, 33]. We obtained similar performance for recognising names as a single category despite not having optimised for this dataset.

There is currently rapid development of the capabilities and use of large language models such as ChatGPT. A main advantage of these methods is that they do what is called zero-shot learning and can perform a variety of tasks without any fine-tuning [34]. They therefore do not require annotated training data for the task at hand and can more quickly adopt to evolving data as well as to adjacent domains. These could be deployed for de-identification purposes but whether they can achieve similar performance as a dedicated model trained for the narrow task at hand would need to be evaluated. Because the output of such models is generated free text, they would require special efforts to integrate in a de-identification solution. For example, care may need to be taken to ensure that the processing does not distort the original text. Computational requirements could also be prohibitive, especially in resource-limited settings.

Annotations and the in-depth evaluation of this study were based on subjective human assessment. While the annotation guideline and framework for the in-depth evaluation were designed to minimise subjectivity, there were difficult cases during annotation involving short tokens that could not be unambiguously classified (despite consultation with native English speakers from the UK, one of whom is a medical doctor), illustrating the fundamental difficulty of the de-identification task. Annotations were performed by four annotators who could consult each other. One limitation of this study is that no formal study of inter-annotator agreement was performed but checks were performed by a second annotator on a random subset.

The target domain for this study is limited in scope: adverse event reports from the UK. These reports are primarily in English and may exhibit UK-specific characteristics. Extending the work to data sets from other countries and languages will be important for the global pharmacovigilance network. Ideally, it will be possible to continually refine a single method across a diverse set of English narratives for use in different contexts, but further studies are required to know if this is the best approach. Pretrained mono- and multi-lingual BERT models exist, which may enable more straightforward adaptation to other languages. In our study, we benefitted from the publicly available i2b2 data set, which is only available in English. However, de-identification methods for electronic health records have successfully been developed for non-English languages such as Italian and Swedish [23, 32] illustrating the feasibility of pre-training and fine-tuning language models such as BERT for de-identification in other languages and datasets. Similarly, future de-identification relying on prompt-based large language models like ChatGPT would already be trained on multiple languages. Alternatively, automated translation to English may allow use of a single de-identification model like the one proposed here.

Our de-identification method can be improved in multiple ways. With more annotated training data or tuning BERT parameters further, BERT could possibly reach even better performance. Additionally, it could be extended to other categories of personal identifiers, some of which may require different, potentially simpler approaches: identifiers like e-mail addresses, ID numbers and phone numbers may for example be better captured using regular expressions. For one record in the Yellow Card test data, BERT failed to recognise part of one doctor’s name preceded by a salutation which may appear obvious to a human and were detected by our regular expressions (see Fig. 5). For user acceptance and trust, straightforward errors like these may be detrimental and use of the ensemble or a similar approach may be considered necessary despite its lower precision. Future research could seek to improve the precision of the ensemble by refining its rules or supplementing them with traditional NLP techniques like part-of-speech tagging. Furthermore, the ensemble could consider probability scores instead of binary labels and/or treat shorter tokens differently than long tokens.

## Conclusions

Automated redaction of names in free-text narratives of adverse event reports using a transformer-based neural network can achieve sufficient recall including shorter tokens like patient initials. In-depth review shows that the rare leaks that occur tend not to compromise patient confidentiality, and precision and false positive rate are acceptable retaining almost all clinically relevant information.

## Electronic supplementary material

Below is the link to the electronic supplementary material.

Supplementary Material 1

Supplementary Material 2

Supplementary Material 3

Supplementary Material 4

Supplementary Material 5

## Data Availability

The 2014 i2b2 dataset analysed during the current study are available in the n2c2 (National NLP Clinical Challenges) repository in the Department of Biomedical Informatics at Harvard Medical School, https://portal.dbmi.hms.harvard.edu/. The Yellow Card data that support the findings of this study are available from the UK Medicines and Health Regulatory Agency (MHRA) but restrictions apply to the availability of these data, which were used under licence for the current study, and so are not publicly available. Data are however available from the authors (contact Eva-Lisa Meldau) upon reasonable request and with permission of the MHRA.

## Footnotes

1 https://yellowcard.mhra.gov.uk/.

2 https://coronavirus-yellowcard.mhra.gov.uk/.

3 https://itunes.apple.com/us/app/apple-store/id990237487?pt=117756671&ct=EYC&mt=8.

4 https://play.google.com/store/apps/details?id=uk.org.mhra.yellowcard.

5 https://prodi.gy/.

## Declarations

### Authors’ information

Uppsala Monitoring Centre is a non-profit foundation established in 1978 through an agreement between the World Health Organisation (WHO) and the Government of Sweden. It is the designated WHO Collaborating Centre for International Drug Monitoring and custodian and manager of VigiBase, the WHO global database of adverse event reports for medicines and vaccines. VigiBase brings together adverse event reports from members of the WHO Programme for International Drug Monitoring including the UK MHRA.

## Abbreviations

BERT: Bidirectional encoder representations from transformers
CRF: Conditional random field
GPT: Generative pre-trained transformer
LSTM: Long short-term memory
MHRA: Medicines and healthcare products regulatory agency
NLP: Natural language processing
UK: United Kingdom
WHO: World Health Organization
YC: Yellow Card
