# Supplementary material for: Automated redaction of names in adverse event reports using transformer-based neural networks
Source: BMC Med Inform Decis Mak. 2024 Dec 23;24:401. doi: 10.1186/s12911-024-02785-9 (PMC11668006; doi:10.1186/s12911-024-02785-9)
Supplement: Supplementary file 1 — Supplementary Material 1 [file 12911_2024_2785_MOESM1_ESM.pdf]

S1 MACHINE-ASSISTED ANNOTATIONS

Prodigy: The machine-assisted annotations with *prodigy* were performed using *prodigy* version 1.10.5. Figure 1 shows how the `ner.teach` recipe was used to identify possible name annotations.

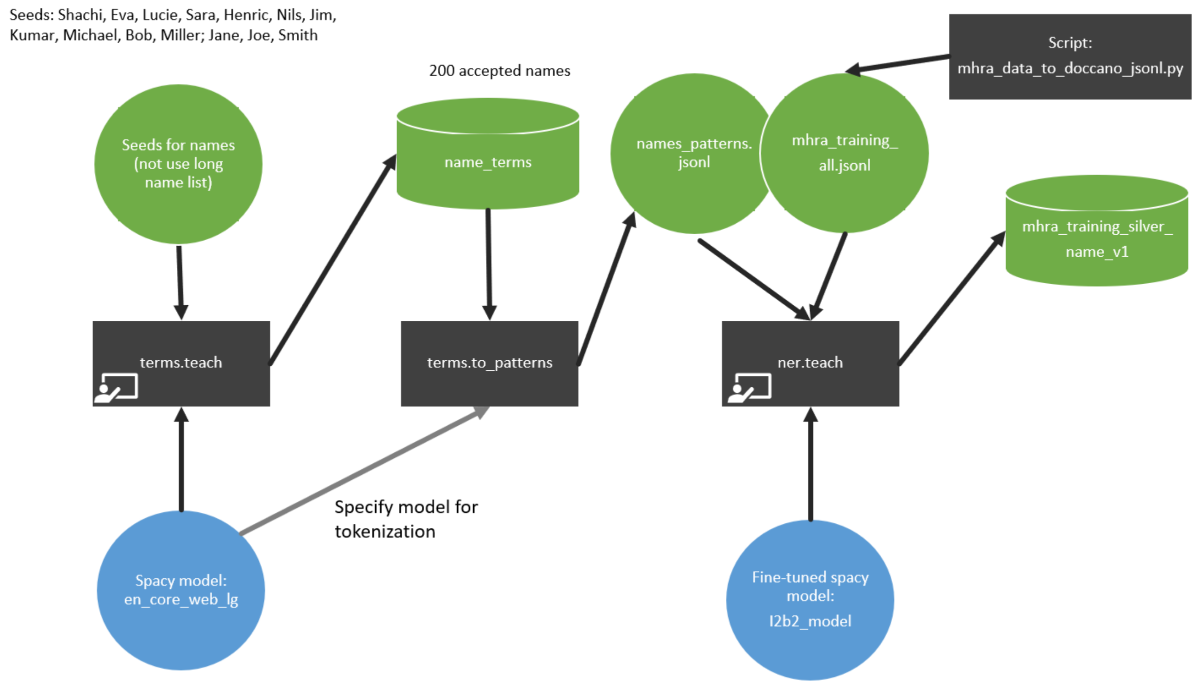

Figure 1 Active learning for NAME category, starting with the creation of name patterns based on seeds, followed by active learning with `ner.teach` recipe. A pre-trained i2b2 model was used in the `ner.teach` recipe.

Commands run:

```
python -m prodigy terms.teach name_terms en_core_web_lg --seeds “Shachi, Eva, Lucie, Sara, Henric, Nils, Jim, Kumar, Michael, Bob, Miller, Jane, Joe, Smith”

python -m prodigy terms.to-patterns name_terms .\data\patterns\name_patterns.jsonl --label NAME --spacy-model en_core_web_lg

python -m prodigy ner.teach mhra_training_silver_name_v1 .\models\i2b2_model .\data\mhra_training\mhra_training_all.jsonl --label NAME --patterns .\data\patterns\name_patterns.jsonl --unsegmented
```

Annotations were done in batches using and the i2b2 model replaced by a fine-tuned model for the Yellow Card data (referred to as *mhr_name_model*), as shown in Figure 2.

Commands run:

```
python -m prodigy train ner mhra_training_silver_name_v1 .\models\i2b2_model --output .\models\mhra_name_model_v1 --eval-split 0.2 --binary
```

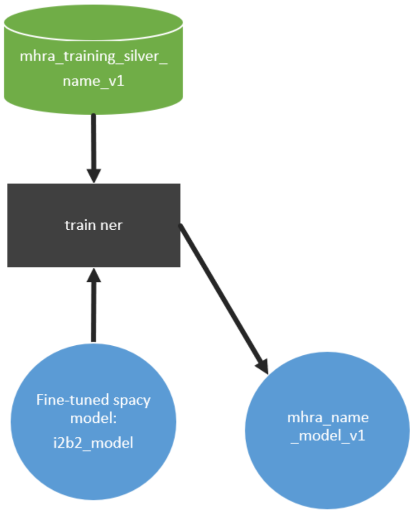

Figure 2 Manually re-training the model within active learning workflow on all previously annotated data. This will be better than the online training in `ner.teach`.

To get the final annotations, the `ner.correct` recipe was used (see Figure 3).

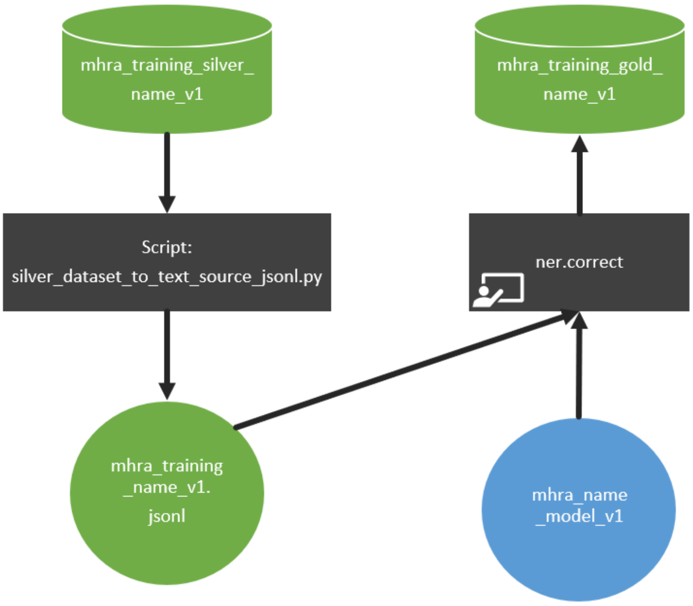

Figure 3 Approach to manually correcting the annotations made by the model.

Commands run:

```
python -m prodigy ner.correct mhra_training_gold_v1 .\models\mhra_name_model_v1 .\data
\mhra_training_name_v1\ mhra_training_name_v1.jsonl --label NAME –unsegmented
```

Initials were manually annotated after they had been identified using regular expressions built from context from identified Yellow Card NAME annotations as well as based on other hand-engineered regular expressions. The workflow is shown in Figure 4.

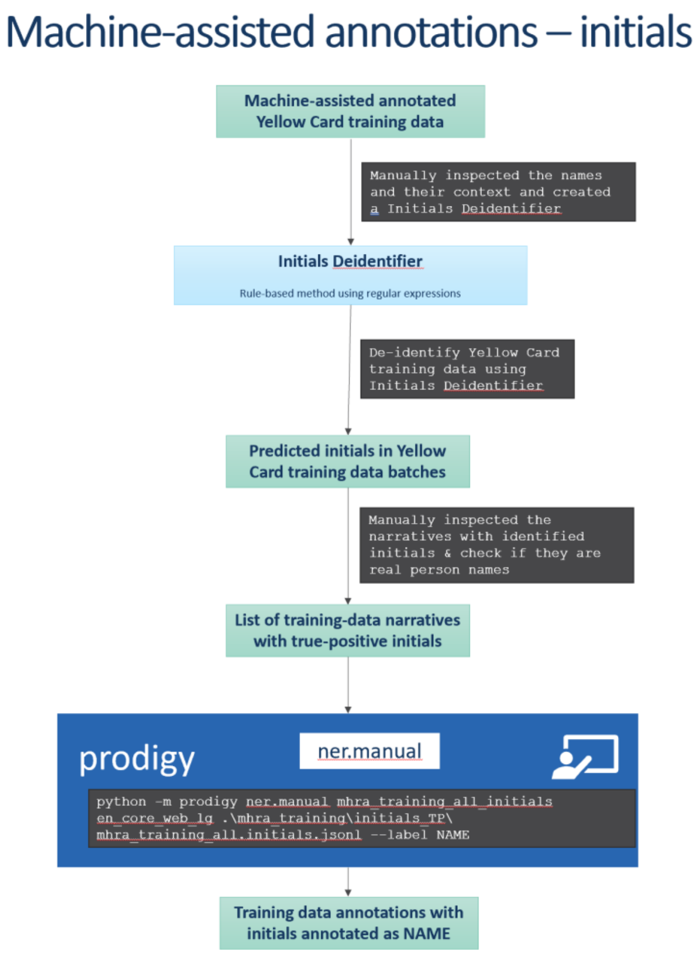

Figure 4 Workflow for machine-assisted annotations of initials.

The code for the regular expressions used in the Initials Deidentifier can be found below:

```
# create regex expresions to search for, and the tag they are mapping to
regex_list: List[Dict[str, Any]] = []
# reusable help strings
typical_initial = r'[A-Z%s]{2,3}[.]?' % self.latin_special_characters_upper_case

# regex rules
# Initials (up til three separate words) following by 'Dr' 'Dr.' 'Doctor' etc
regex_list.append({'tag':TagType.DOCTOR, 'expression':r'(?<=(\b(dr|DR|Dr)[^\w]))%s(?=(\b))' %
typical_initial, 'flags':0})
regex_list.append({'tag':TagType.DOCTOR, 'expression':r'(?<=(\b(dr|DR|Dr)[^\w][^\w]))%s(?=(\b))' %
typical_initial, 'flags':0})
regex_list.append({'tag':TagType.DOCTOR, 'expression':r'(?<=((doctor|Doctor|DOCTOR)[^\w]))%s(?=(\b))'
% typical_initial, 'flags':0})
regex_list.append({'tag':TagType.DOCTOR,
'expression':r'(?<=((doctor|Doctor|DOCTOR)[^\w][^\w]))%s(?=(\b))' % typical_initial, 'flags':0})
# Initials (up til three separate words) followed by 'MD' 'M.D.' ',MD' etc
regex_list.append({'tag':TagType.DOCTOR, 'expression':r'(?<=(\b))%s(?=([^\w](MD|md)\b))' %
typical_initial, 'flags':0})
```

```
regex_list.append({'tag':TagType.DOCTOR, 'expression':r'(?<=(\b))%s(?=([^\w](M[^\w]D|m[^\w]d)\b))' %
typical_initial, 'flags':0})
regex_list.append({'tag':TagType.DOCTOR, 'expression':r'(?<=(\b))%s(?=([^\w][^\w](MD|md)\b))' %
typical_initial, 'flags':0})
regex_list.append({'tag':TagType.DOCTOR,
'expression':r'(?<=(\b))%s(?=([^\w][^\w](M[^\w]D|m[^\w]d)\b))' % typical_initial, 'flags':0})
# Initials (up til three separate words) following by 'Mr' 'Mr.' 'PT:' 'Patient:' 'Name:' etc
regex_list.append({'tag':TagType.PATIENT, 'expression':r'(?<=(\b(mr|MR|Mr|ms|MS|Ms)[^\w]))%s(?=(\b))'
% typical_initial, 'flags':0})
regex_list.append({'tag':TagType.PATIENT,
'expression':r'(?<=(\b(mr|MR|Mr|ms|MS|Ms)[^\w][^\w]))%s(?=(\b))' % typical_initial, 'flags':0})
regex_list.append({'tag':TagType.PATIENT,
'expression':r'(?<=(\b(mrs|MRS|Mrs|pt:|PT:|Pt:)[^\w]))%s(?=(\b))' % typical_initial, 'flags':0})
regex_list.append({'tag':TagType.PATIENT, 'expression':r'(?<=(\b(mrs|MRS|Mrs)[^\w][^\w]))%s(?=(\b))' %
typical_initial, 'flags':0})
regex_list.append({'tag':TagType.PATIENT, 'expression':r'(?<=(\b(miss|MISS|Miss)[^\w]))%s(?=(\b))' %
typical_initial, 'flags':0})
regex_list.append({'tag':TagType.PATIENT,
'expression':r'(?<=(\b(miss|MISS|Miss)[^\w][^\w]))%s(?=(\b))' % typical_initial, 'flags':0})
regex_list.append({'tag':TagType.PATIENT, 'expression':r'(?<=(\b(name:|NAME:|Name:)[^\w]))%s(?=(\b))'
% typical_initial, 'flags':0})
regex_list.append({'tag':TagType.PATIENT,
'expression':r'(?<=(\b(patient:|PATIENT:|Patient:)[^\w]))%s(?=(\b))' % typical_initial, 'flags':0})

# Initials after certain words
regex_list.append({'tag':TagType.PATIENT, 'expression':r'(?<=(\b(asked ))%s(?=(\b))' %
typical_initial, 'flags':0})
regex_list.append({'tag':TagType.PATIENT, 'expression':r'(?<=(\b(talked with ))%s(?=(\b))' %
typical_initial, 'flags':0})
regex_list.append({'tag':TagType.PATIENT, 'expression':r'(?<=(\b(discharging nurse ))%s(?=(\b))' %
typical_initial, 'flags':0})
regex_list.append({'tag':TagType.PATIENT, 'expression':r'(?<=(\b(Note added by: ))%s(?=(\b))' %
typical_initial, 'flags':0})
regex_list.append({'tag':TagType.PATIENT, 'expression':r'(?<=(\b(mother ))%s(?=(\b))' %
typical_initial, 'flags':0})
regex_list.append({'tag':TagType.PATIENT, 'expression':r'(?<=(\b(daughter ))%s(?=(\b))' %
typical_initial, 'flags':0})
regex_list.append({'tag':TagType.PATIENT, 'expression':r'(?<=(\b(speak to ))%s(?=(\b))' %
typical_initial, 'flags':0})
regex_list.append({'tag':TagType.PATIENT, 'expression':r'(?<=(\b(I told ))%s(?=(\b))' %
typical_initial, 'flags':0})
# When we ran this script in December 2022, there was a typo in this rule "informatio" instead of
"information" which means that this rule did not work propoerly
regex_list.append({'tag':TagType.PATIENT, 'expression':r'(?<=(\b(information was provided by
))%s(?=(\b))' % typical_initial, 'flags':0})
regex_list.append({'tag':TagType.PATIENT, 'expression':r'(?<=(\b(Reaction description: ))%s(?=(\b))'
% typical_initial, 'flags':0})
```

```
regex_list.append({'tag':TagType.PATIENT, 'expression':r'(?<=(\b(Dear )))%s(?=(\b))' %
typical_initial, 'flags':0})
regex_list.append({'tag':TagType.PATIENT, 'expression':r'(?<=(\b(Hi )))%s(?=(\b))' % typical_initial,
'flags':0})

# Initials before certain words
regex_list.append({'tag':TagType.PATIENT, 'expression':r'(?<=(\b))%s(?=(\b(told me)))' %
typical_initial, 'flags':0})
regex_list.append({'tag':TagType.PATIENT, 'expression':r'(?<=(\b))%s(?=(\b(has noted)))' %
typical_initial, 'flags':0})
regex_list.append({'tag':TagType.PATIENT, 'expression':r'(?<=(\b))%s(?=(\b(complained of)))' %
typical_initial, 'flags':0})
regex_list.append({'tag':TagType.PATIENT, 'expression':r'(?<=(\b))%s(?=(\b(complained about)))' %
typical_initial, 'flags':0})
regex_list.append({'tag':TagType.PATIENT, 'expression':r'(?<=(\b))%s(?=(\b(developed)))' %
typical_initial, 'flags':0})
regex_list.append({'tag':TagType.PATIENT, 'expression':r'(?<=(\b))%s(?=(\b(presented for)))' %
typical_initial, 'flags':0})
regex_list.append({'tag':TagType.PATIENT, 'expression':r'(?<=(\b))%s(?=(\b(feels)))' %
typical_initial, 'flags':0})
regex_list.append({'tag':TagType.PATIENT, 'expression':r'(?<=(\b))%s(?=(\b(was prescribed)))' %
typical_initial, 'flags':0})
regex_list.append({'tag':TagType.PATIENT, 'expression':r'(?<=(\b))%s(?=(\b(started getting)))' %
typical_initial, 'flags':0})
regex_list.append({'tag':TagType.PATIENT, 'expression':r'(?<=(\b))%s(?=(\b(stopped getting)))' %
typical_initial, 'flags':0})
regex_list.append({'tag':TagType.PATIENT, 'expression':r'(?<=(\b))%s(?=(\b(had developed)))' %
typical_initial, 'flags':0})
regex_list.append({'tag':TagType.PATIENT, 'expression':r'(?<=(\b))%s(?=(\b(was discharged)))' %
typical_initial, 'flags':0})
regex_list.append({'tag':TagType.PATIENT, 'expression':r'(?<=(\b))%s(?=(\b(ha[d,s] received)))' %
typical_initial, 'flags':0})
regex_list.append({'tag':TagType.PATIENT, 'expression':r'(?<=(\b))%s(?=(\b(was given)))' %
typical_initial, 'flags':0})
regex_list.append({'tag':TagType.PATIENT, 'expression':r'(?<=(\b))%s(?=(\b(started taking)))' %
typical_initial, 'flags':0})

# Initials between certain words
regex_list.append({'tag':TagType.PATIENT, 'expression':r'(?<=(\b(observe)))%s(?=(\b(for)))' %
typical_initial, 'flags':0})
regex_list.append({'tag':TagType.PATIENT, 'expression':r'(?<=(\b(asked)))%s(?=(\b(to)))' %
typical_initial, 'flags':0})


# search for all regex expressions and save to tag_list and character_tag_map
for regex in regex_list:
    for match in re.finditer(regex['expression'], narrative.text, flags=regex['flags']):
        self._create_tag_from_match(match_object=match, narrative=narrative, tag_type=regex['tag'])
regex_list = []
```
