# Supplementary material for: Automated redaction of names in adverse event reports using transformer-based neural networks
Source: BMC Med Inform Decis Mak. 2024 Dec 23;24:401. doi: 10.1186/s12911-024-02785-9 (PMC11668006; doi:10.1186/s12911-024-02785-9)
Supplement: Supplementary file 2 — Supplementary Material 2 [file 12911_2024_2785_MOESM2_ESM.pdf]

S2 ANNOTATION GUIDELINE

Introduction

This guideline indicates what we consider to be a person NAME, and how we should annotate them. These can be different types of person names, e.g., patients, doctors, other healthcare professionals, relatives, guardians etc.

Additional Instructions

- If you find narratives written in a language other than English, make a note of the row number and do not label it (even if you understand the language).

Names

- Contiguous forenames and surnames should be labelled together
- Titles (mrs, mr, dr, miss, ms, MD, PhD etc) should not be labelled
- Name punctuation (the dot in John H. Smith, the - in Sara-Lisa) should be labelled
- Initials should be labelled as single tokens
- The “s” in the possessive form (“John’s”) should not be labelled.
- Author names in citation of publications should not be labelled.

John H. Smith went to buy a newspaper. Mr Smith knew he had found the right one because it had JHS written in the top right corner. He was in Dr Muller’s care.
