# Supplementary material for: Automated redaction of names in adverse event reports using transformer-based neural networks
Source: BMC Med Inform Decis Mak. 2024 Dec 23;24:401. doi: 10.1186/s12911-024-02785-9 (PMC11668006; doi:10.1186/s12911-024-02785-9)
Supplement: Supplementary file 3 — Supplementary Material 3 [file 12911_2024_2785_MOESM3_ESM.pdf]

S3 BERT METHOD

Below is some python code showing the implementation of the BERT method using tensorflow and the choice of parameters:

```
from transformers import AutoTokenizer
from transformers import TFAutoModelForTokenClassification
from tensorflow.keras.losses import CategoricalCrossentropy
from tensorflow.keras.optimizers import Adam
from tensorflow import sigmoid
import numpy as np

# Tokenizer
checkpoint = "bert-base-uncased"
tokenizer = AutoTokenizer.from_pretrained(checkpoint)

number_classes = 2  # NAME, NON-NAME
model = TFAutoModelForTokenClassification.from_pretrained(
    tokenizer.checkpoint, num_labels=number_classes + 1
)


# Training parameters
epochs = 5
batch_size = 1
max_sequence_length = 510 # longer narratives are split by a custom function -
not shown here

loss = CategoricalCrossentropy(from_logits=True)
optimizer = Adam(learning_rate=1e-5)

model.compile(
    optimizer=optimizer,
    loss=loss,
)

# Training function
model.fit(
    x=x_train,
    y=y_train,
    epochs=epochs,
    shuffle=False,
    validation_data=(x_val, y_val, sample_weights_val),
    sample_weight=sample_weights_train,
    batch_size=batch_size
)


# De-identify using model predictions and threshold
threshold = 0.9 # only predict NONNAME if probability for class > threshold
```

```
model_output = model.predict(
    x=x_test,
    batch_size=batch_size,
)

class TagType(Enum):
    """A class defining tag objects and wwther or not they are safe."""
    NONNAME = (0, "NONNAME")
    NAME = (1, "NAME")

type_number_mapping = {
    TagType.NONNAME: 0,
    TagType.NAME: 1
}

all_narratives_probabilities =
sigmoid(model_output.logits[:,1:max_sequence_length+1,:])

for narrative_index, narrative in enumerate(split_narratives):
    narrative_probabilities = all_narratives_probabilities[narrative_index]
    # De-identify depending on probabilities and threshold
    tokens = narrative.get_token_list(tokenizer=tokenizer)
    for token_index, token in enumerate(tokens):
        if
narrative_probabilities[token_index][type_number_mapping[TagType.NONNAME]] >
threshold:
            predicted_class = TagType.NONNAME
        else:
            predicted_class = TagType.NAME
        narrative.create_tag_for_token(token=token, tag_type=predicted_class,
overwrite=True)


# Sample weight computation

def prepare_sample_weights(self, targets: np.ndarray) -> np.ndarray:
        """
        Computes and returns the sample weight array.

        The sample weights are used to pad the sequence. The padded entries
get the weight 0 and will thereby not influence
        the output and training. The sample weights are further used to
balance the classes, giving higher weights to
        less represented classes. The number of samples per class are taken
from number_samples_per_class.
        :param targets: list with the targets as padded, scalar values
        :return: matrix with weight per token of each narrative
        """
```

```python
    print("Entering prepare_sample_weights...")
    class_weights = np.zeros((self.number_classes + 1,))

    total_samples = sum(self.number_samples_per_class.values())

    for label, class_samples in self.number_samples_per_class.items():
        value = total_samples / class_samples

        weight = 1 # default weight

        if value > 10000:
            weight = 64
        elif value > 1000:
            weight = 16
        elif value > 100:
            weight = 4
        elif value > 50:
            weight = 2

        class_weights[label] = weight

    sample_weights = np.take(class_weights, targets.astype(np.int64))

    return sample_weights
```
