# Supplementary material for: Automated redaction of names in adverse event reports using transformer-based neural networks
Source: BMC Med Inform Decis Mak. 2024 Dec 23;24:401. doi: 10.1186/s12911-024-02785-9 (PMC11668006; doi:10.1186/s12911-024-02785-9)
Supplement: Supplementary file 4 — Supplementary Material 4 [file 12911_2024_2785_MOESM4_ESM.pdf]

S4 RULE-BASED METHOD

First, four base name forms are defined:

- Capital word (A0): A capital letter followed by one or more lowercase letters.
- Initials (A1): A capital letter optionally followed by a period (for e.g., “G.” or “G”).
- Name succeeding a chunk of text (B1): Any word optionally followed by an initial (A1) or a capitalised word (A0), optionally followed by a capital word (A1) (for e.g., “M, Fuente”, “M. Fuente”, “fuente”, “marcus Fuente”, “M., Fuente”, “M.”, “Johnny B. Goode”).
- Name preceding a chunk of text (B2): A mirror of rule (B1) where the anchor is at the end of the pattern, any word preceded optionally by an initial (A1) or a capital word (A0) optionally preceded by a capital word (A0).

Different sections of the name forms may be optionally delimited by commas.

The rule-based classifier identifies names given in the base form identified by common prefixes and titles based on these rules

We use the base forms to compose the rules for extracting the base forms by matching common prefixes, suffixes and titles commonly seen in clinical text which are illustrated below:

1. The string “dr”, “dr.” or “doctor” (case-insensitive) followed by (B1).
2. The string “mr”, “ms”, “mrs”, “miss”, “pt:”, “name:” or “patient:” (case-insensitive) followed by (B1).
3. (B2) followed by the string “md”, “m.d.”, “,md” (case-insensitive).

Corresponding python code:

```
# Name (up to three separate words) following by 'Dr' 'Dr.' 'Doctor' etc
regex_list = []
regex_list.append(
    {
        "expression": r"(?<=(\b(dr|DR|Dr)[^\w]))%s(?=(\b))"
        % typical_name_following_something,
        "flags": 0,
    }
)
regex_list.append(
    {
        "expression": r"(?<=(\b(dr|DR|Dr)[^\w][^\w]))%s(?=(\b))"
        % typical_name_following_something,
        "flags": 0,
    }
)
regex_list.append(
    {
        "expression": r"(?<=((doctor|Doctor|DOCTOR)[^\w]))%s(?=(\b))"
        % typical_name_following_something,
        "flags": 0,
    }
```

```
)
regex_list.append(
    {
        "expression": r"(?<=((doctor|Doctor|DOCTOR)[^\w][^\w]))%s(?=(\b))"
        % typical_name_following_something,
        "flags": 0,
    }
)
# Name (up to three separate words) followed by 'MD' 'M.D.' ',MD' etc
regex_list.append(
    {
        "expression": r"(?<=(\b))%s(?=([^\w](MD|md)\b))"
        % typical_name_preceiding_something,
        "flags": 0,
    }
)
regex_list.append(
    {
        "expression": r"(?<=(\b))%s(?=([^\w](M[^\w]D|m[^\w]d)\b))"
        % typical_name_preceiding_something,
        "flags": 0,
    }
)
regex_list.append(
    {
        "expression": r"(?<=(\b))%s(?=([^\w][^\w](MD|md)\b))"
        % typical_name_preceiding_something,
        "flags": 0,
    }
)
regex_list.append(
    {
        "expression": r"(?<=(\b))%s(?=([^\w][^\w](M[^\w]D|m[^\w]d)\b))"
        % typical_name_preceiding_something,
        "flags": 0,
    }
)
# Name (up to three separate words) following by 'Mr' 'Mr.' 'PT:' 'Patient:' 'Name:' etc
regex_list.append(
    {
        "expression": r"(?<=(\b(mr|MR|Mr|ms|MS|Ms)[^\w]))%s(?=(\b))"
        % typical_name_following_something,
        "flags": 0,
    }
)
regex_list.append(
    {
        "expression": r"(?<=(\b(mr|MR|Mr|ms|MS|Ms)[^\w][^\w]))%s(?=(\b))"
```

```python
        % typical_name_following_something,
        "flags": 0,
    }
)
regex_list.append(
    {
        "expression": r"(?<=(\b(mrs|MRS|Mrs|pt:|PT:|Pt:)[^\w]))%s(?=(\b))"
        % typical_name_following_something,
        "flags": 0,
    }
)
regex_list.append(
    {
        "expression": r"(?<=(\b(mrs|MRS|Mrs)[^\w][^\w]))%s(?=(\b))"
        % typical_name_following_something,
        "flags": 0,
    }
)
regex_list.append(
    {
        "expression": r"(?<=(\b(miss|MISS|Miss)[^\w]))%s(?=(\b))"
        % typical_name_following_something,
        "flags": 0,
    }
)
regex_list.append(
    {
        "expression": r"(?<=(\b(miss|MISS|Miss)[^\w][^\w]))%s(?=(\b))"
        % typical_name_following_something,
        "flags": 0,
    }
)
regex_list.append(
    {
        "expression": r"(?<=(\b(name:|NAME:|Name:)[^\w]))%s(?=(\b))"
        % typical_name_following_something,
        "flags": 0,
    }
)
regex_list.append(
    {
        "expression": r"(?<=(\b(patient:|PATIENT:|Patient:)[^\w]))%s(?=(\b))"
        % typical_name_following_something,
        "flags": 0,
    }
)

# search for all regex expressions
```
