# Supplementary material for: Automated redaction of names in adverse event reports using transformer-based neural networks
Source: BMC Med Inform Decis Mak. 2024 Dec 23;24:401. doi: 10.1186/s12911-024-02785-9 (PMC11668006; doi:10.1186/s12911-024-02785-9)
Supplement: Supplementary file 5 — Supplementary Material 5 [file 12911_2024_2785_MOESM5_ESM.pdf]

S5 EVALUATION GUIDELINE

Introduction

This guideline describes the process of evaluating the automatically anonymized narratives. It also aims to give some practical examples of points which should be considered when conducting the evaluations.

Background

A BERT-based algorithm is used to de-identify every personal identifier, such as names, from ADRs narratives (see Annotation Guideline).

Domain experts made manual annotations on a set of thousands of narratives from cases reported to the MHRA. These annotations highlighted any personal identifier (e.g., names, dates, locations, IDs, etc.) that was present in the narratives text. The compiled body of annotations constituted the Golden Standard for the evaluation described herein.

Evaluation process

For the present evaluation, only the personal identifiers NAMEs were masked for the evaluators.

1.1 When presented with the de-identified narrative text, the evaluator must answer the following question regarding the usefulness of the de-identified narratives. The evaluator will not reveal the masked information so he/she should answer based on their perception of what information could be hidden behind the mask:

- Do you think this narrative is missing information to assess the case and such information could be behind the mask?
- *The answer should be YES if: there are pieces of information missing in the text and the masks could possibly hide such information. The narratives will not always contain all details from the full case originally reported which are necessary to conduct a causality assessment or other safety assessments. However, the evaluator will have to judge from the information that seems to be available altogether.*
- *The answer should be NO if: The assessor is certain the masks are hiding patient, Healthcare Professional, or others' names. It should also be NO if the masked information appears to be irrelevant to understand the whole narrative from an assessor's perspective.*
- *See examples in section 4.*

1.2 When the masks are removed and the full text is shown, the evaluator must answer the following question regarding the information revealed:

- Did the information removed have an impact on the assessment of the case?
- *Examples of information masked that could have an impact on the assessment of the case:*
  - *Treatments, adverse event terminology, outcomes from adverse events, diagnoses that shed some light on causality assessment, and any other information on the clinical course of events.*
- *See examples in section 4.*

1.3 The evaluator must consider any personal identifiers (i.e., names, for the purpose of this evaluation) that were not picked by the de-identification algorithm and were therefore not masked. Such “leaked” information will be classified as:

**NAME Category**	**Definition**	**Examples**
Directly identifiable	A person can **most likely** be identified by using such token.	Full names (also as part of email addresses):<br>- **Eva-Lisa Meldau**<br>- **carlos.melgarejo.gonzalez***@email.com*
Indirectly identifiable	These names alone are not enough to identify a subject. However, in combination with context or additional identifiers present in the narrative (e.g. telephone number, occupational details, memberships or affiliations, etc.), the subject’s identity might be given away.	- Mr. **Barrett**<br>- Dr. **Bista**<br>- D. **Jones** (common surname) + LPCH ID<br>- **Joe** (common name) + workplace, telephone, and medical centre.
No-identifiable	Identifying the subject with this information is **highly unlikely**.	- Initials: *“**J** was taken to the hospital”*<br>- Few letters of name left unmasked: *“The patient* [redacted] [redacted]***ra*** *was taken to the hospital”*<br>- Very common names*: *“**Jack** was taken to the hospital”*<br><br>*Only names that are very common in narrative’s respective countries should be classified here (the UK in this case). The slightest hesitation from the evaluator should point to choosing “indirectly identifiable”.

It should be noted that more levels of identifiability could be drawn, and the assessor may find a given token more identifiable than others within the same category.

Practical examples

The following fictional narrative text was created to illustrate some instances that the evaluator might encounter.

Masked sample.

> [redacted] *was taken to A&E after fainting. Mr J* [redacted] *10 stones during the previous year, family and GP were much concerned. Attended by Dr.* [redacted]*, Dr.* [redacted] *and Dr.* [redacted] *at St.* [redacted] *Hospital, Sheffield.* [redacted] *had previous history of headaches and malignant hypertension. However, lab results showed concerning* [redacted] *values of Rixivia plasma concentration and Hb.* [redacted] *surgery performed in recent years.* [redacted]*,* [redacted]*,* [redacted] [redacted] *paper consulted for evidence on patient's condition in the literature. Patient had been started on Rixivia and* [redacted] *following latest surgery. Patient discharged later that day, thought to be* [redacted].

Unmasked sample.

> ***Joe Jones*** *was taken to A&E after fainting. Mr* ***J haslost*** *10 stones during the previous year, family and GP were much concerned. Attended by Dr.* ***Barrett****, Dr.* ***Mendau*** *and Dr.* ***Bista*** *at St.* ***Georges*** *Hospital, Sheffield.* ***Jones*** *had previous history of headaches and malignant hypertension. However, lab results showed concerning* ***nadir*** *values of Rixivia plasma concentration and Hb.* ***Ivor Lewis*** *surgery performed in recent years.* ***D. Xieng, A. Amela, P. Smith*** *paper consulted for evidence on patient's condition in the literature. Patient had been started on Rixivia and* ***FancyDrug*** *after last surgery. Patient discharged later that day, thought to be* ***vasovagal****.*

Considerations:

- <u>Leaked names</u>: In the sample text there is an instance of a leaked name that was not picked up by the de-identifier algorithm, the initial "**J**" for Joe Jones. Such leaked information would be classified as "**no-identifiable**" since no other personal identifiers that may clue into J's identity are shown in the narrative and many subjects can go by the initial J (even knowing Sheffield was the location).

- <u>Irrelevant names masked:</u> Many hospitals and healthcare centres have people's names and may or may not be picked by the algorithm. Similarly, some narratives may include names of authors of publication or the like. Since this information is neither relevant for the assessment of the narrative nor can be considered a NAME, it is indifferent whether it is masked or not by the algorithm (this would not apply should other personal identifiers be considered for assessment).

- <u>Relevant information mistakenly masked</u>: In the text we find some instances of text that has mistakenly been taken for a name. Firstly, the word "**haslost**" is a misspelling of "has lost". The fact that the patient has experienced a major weight

loss during the previous year is not understood when “haslost” is masked. Hence, we can deem it relevant for the narrative assessment.

The second instance of this kind is the masking of the word “**nadir**”. In this case nadir refers to the trough level of Haemoglobin and the made-up drug Rixivia.

“**Ivor Lewis**” is the name of a type of esophagectomy, which gives us more context on the patient’s medical history and the interventions he went through.

Masking the fictional drug name “**FancyDrug**” hides the only available information in the narrative of the other drug the patient was taking concomitant to Rixivia. Assuming we have no detail of such medication in the report, this information is relevant.

“**Vasovagal**” is the only mention of a diagnose we find in the narrative. It refers to a vasovagal response, which was deemed the cause of the patient’s condition and justified his discharge.

Assessment outcome according to points stated above:

- Question 3.1 → YES: Before unmasking the narrative, it is obvious that some important information is missing behind the masks and such information will help us understand the patient’s clinical course when unmasked.
- Question 3.2 → YES: As seen in the considerations above, some of the information removed by masking was important to understand the narrative (i.e., “relevant information mistakenly masked”)
- Question 3.3 → Mr J can be classified as “**No-identifiable**” even though we have information about the hospital he attended being in Sheffield, which is a large city with many hospitals that could be the one behind the mask. Even if we happened to know the name of the hospital in this case, the initial “J” would not be enough to identify the patient since many patients could go by such initial. Only a location with a really small population and the presence of additional identifiers in the narrative would have made “Mr J” an “indirectly identifiable” token in this case.
